# Leveraging Joint Mechanics Simplifies the Neural Control of Movement

**DOI:** 10.3389/fnint.2022.802608

**Published:** 2022-03-21

**Authors:** Daniel Ludvig, Mariah W. Whitmore, Eric J. Perreault

**Affiliations:** ^1^Department of Biomedical Engineering, Northwestern University, Evanston, IL, United States; ^2^Shirley Ryan AbilityLab, Chicago, IL, United States; ^3^Department of Physical Medicine and Rehabilitation, Northwestern University, Chicago, IL, United States

**Keywords:** joint mechanics, mechanical impedance, neural control, muscle activation, perceived difficulty, task performance

## Abstract

Behaviors we perform each day, such as manipulating an object or walking, require precise control of the interaction forces between our bodies and the environment. These forces are generated by muscle contractions, specified by the nervous system, and by joint mechanics, determined by the intrinsic properties of the musculoskeletal system. Depending on behavioral goals, joint mechanics might simplify or complicate control of movement by the nervous system. Whether humans can exploit joint mechanics to simplify neural control remains unclear. Here we evaluated if leveraging joint mechanics simplifies neural control by comparing performance in three tasks that required subjects to generate specified torques about the ankle during imposed sinusoidal movements; only one task required torques that could be generated by leveraging the intrinsic mechanics of the joint. The complexity of the neural control was assessed by subjects’ perceived difficulty and the resultant task performance. We developed a novel approach that used continuous estimates of ankle impedance, a quantitative description of the joint mechanics, and measures of muscle activity to determine the mechanical and neural contributions to the net ankle torque generated in each task. We found that the torque resulting from changes in neural control was reduced when ankle impedance was consistent with the task being performed. Subjects perceived this task to be easier than those that were not consistent with the impedance of the ankle and were able to perform it with the highest level of consistency across repeated trials. These results demonstrate that leveraging the mechanical properties of a joint can simplify task completion and improve performance.

## Introduction

Completing motor tasks that require contact is dependent on an ability to regulate the relationship between limb motions and interaction forces with the environment. The nature of this relationship depends on the requirements of each task. For example, continuous hopping requires joint torques that increase sufficiently upon impact to launch the hopper into the air (Farris and Sawicki, 2012), whereas landing from a jump requires a decrease in joint torques after the initial impact to cease motion and stabilize the body (Decker et al., 2003). The flexibility to perform these similar but contrasting actions arises from our ability to coordinate joint motions and torques across a range of functionally relevant situations.

Two strategies for coordinating limb motions and interaction forces are leveraging the mechanical properties of the limb associated with the current state of the neuromuscular system, and actively regulating joint torques or motions by changing the state of the neuromuscular system as can occur through changes in muscle activation. The mechanical properties of a limb or joint are often quantified by estimates of impedance, the dynamic relationship between imposed motions and the resulting torques (Hogan, 1985b). By setting impedance to a desired value, it is possible to achieve a variety of motion-torque relationships, though these are of course limited to relationships that are physiologically plausible. For example, the static component of limb impedance—often referred to as stiffness—serves to generate torques that oppose externally applied motions. Impedance control might therefore be sufficient for hopping, in which joint torques must increase with increasing joint excursion (Farris and Sawicki, 2012). However, for landing from a jump in which joint torques tend to decrease—following an initial increase—with increasing joint excursion (Decker et al., 2003), an impedance control strategy alone would not be possible. The alternative is to change muscle activity continuously throughout the task to achieve the desired motion-torque relationship; this is the only feasible solution when the impedance established by the current state of the neuromuscular system is not sufficient for the demands of the task being performed.

There are many conditions in which impedance regulation provides a simple and effective control strategy for stabilizing limb posture or movement trajectories. Impedance has been shown to be regulated in many postural tasks (Finley et al., 2012; Krutky et al., 2013; Zenzeri et al., 2014), often to stabilize the human limb against unpredictable disturbances. These adaptations can occur through changes in limb configuration (Mussa-Ivaldi et al., 1985; Tsuji et al., 1995; Trumbower et al., 2009; Krutky et al., 2013), volitional muscle activation (Hogan, 1984; De Serres and Milner, 1991; Mirbagheri et al., 2000), or involuntary activation through reflex pathways (Sinkjaer et al., 1988; Doemges and Rack, 1992; Mirbagheri et al., 2000; van der Helm et al., 2002). The same mechanisms can be used to stabilize a limb during movement (Burdet et al., 2001; Franklin et al., 2007; Ludvig et al., 2017). However, it remains unknown whether a similar strategy of impedance regulation would be advantageous for the coordination of motion and torque when stability is not a primary concern.

Impedance control is not a viable strategy for coordinating torque with motion when task demands are not compatible with the intrinsic mechanical properties of a joint or limb. In these cases, it is necessary to regulate joint torques through changes in muscle activation. This approach has the advantage of being flexible enough to generate arbitrary torque profiles independent of the movement of the joint, even profiles that are inconsistent with the inherent mechanical properties of the joint, such as those that emulate a negative stiffness. However, such a strategy may require more complex neural control and associated decreases in performance or increases in cognitive demand relative to an impedance control strategy. These complexities may arise from external factors such as the unpredictable mechanical properties of the environment (Johansson and Westling, 1988), or internal factors such as the non-linear length-tension (Gordon et al., 1966) and force-velocity (Hill, 1938; Wickiewicz et al., 1984) properties of muscle or the inherent noisiness of muscle activation (Carlton et al., 1985; Harris and Wolpert, 1998; Jones et al., 2002; Tracy et al., 2005). Despite these potential disadvantages, controlling torque through changes in muscle activation could be more intuitive, and may result in tasks that are perceived to be easy and can be completed accurately (Corbett et al., 2011). Thus, it remains unclear which strategy would be more advantageous in terms of neural simplicity, task performance and metabolic cost.

The purpose of this study was to determine if humans leverage the impedance of a limb to complete a motor task more simply, which we defined as perceived to be easier and more consistent, when that impedance is aligned with task demands. We evaluated this by determining how the strategy chosen by the subject influenced the perception of difficulty and task performance. All experiments were performed on the human ankle. Subjects were required to complete three tasks differing in the required coordination between ankle motions and ankle torques. Specifically, the three tasks had different slopes associated with their motion-torque profiles. These slopes have been described as the “quasi-stiffness” of a joint, as they characterize a spring-like behavior that can be different from the actual mechanical properties of the joint (Latash and Zatsiorsky, 1993; Rouse et al., 2013). One of the tasks had a positive quasi-stiffness consistent with the physiological impedance of the ankle, allowing it to be completed either by leveraging the impedance of the ankle or actively regulating ankle torques through changes in muscle activation. The other two tasks had zero or negative quasi-stiffness and could only be completed by explicitly regulating ankle torques through changes in muscle activation, allowing us to evaluate the influence of this strategy on task performance. We computed ankle impedance continuously throughout the experiment while simultaneously measuring the activity of the major muscles crossing the ankle. These measures were used to determine the contributions of ankle impedance and changes in neural control to the net ankle torque. We expected that when impedance of the ankle was consistent with the task requirements there would be less ankle torque due to muscle activity. We hypothesized that this increased reliance on joint impedance and lesser reliance on cyclic muscle activation would result in a task that was perceived easier and more consistent to perform. These results clarify the conditions in which impedance control is used and demonstrate the impact of that use on task difficulty and performance.

Portions of this work have been previously presented in abstract form (Ludvig et al., 2020).

## Materials and Methods

### Ethical Approval

Twenty unimpaired adults (7 female, 13 male; 27 ± 3 years) participated in this study. All subjects provided informed consent to the protocol, which was approved by the Northwestern University Institutional Review Board.

### Apparatus

**FIGURE 1:**
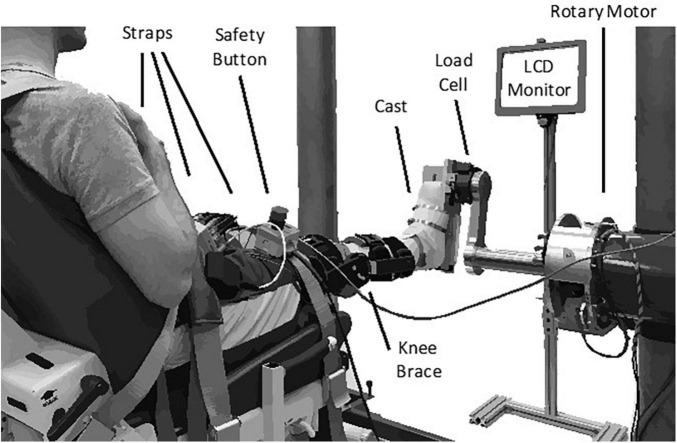
Experimental apparatus.

We secured each subject’s right ankle to an electric rotary motor (BSM90N-3150AF, Baldor, Fort Smith, AR) via a custom fiberglass cast (Figure 1). The cast encased the entire foot but did not cover the ankle joint, preserving full range-of-motion. We aligned the ankle to the center of rotation of the motor and restricted movement to the sagittal plane. Subjects sat reclined with their hips at 135 deg and their right leg extended in front of them. We fixed the right knee at 15 deg of flexion using a brace (Innovator DLX, Össur, Reykjavik, Iceland) and secured it, along with the torso, to the chair using straps. We recorded the ankle angle using an encoder integrated with the motor. We used a 6-degree-of-freedom load cell (45E15A4, JR3, Woodland, CA, United States) to acquire force and torque data about the ankle. We controlled the motor using a position control scheme, so the position of the subject’s ankle was always dictated by the position of the motor.

We measured electromyograms (EMGs) from four muscles crossing the ankle—medial and lateral gastrocnemius (MG and LG), soleus (SOL), and tibialis anterior (TA). Measurements were made using bipolar surface electrodes (Noraxon 272, Scottsdale, AZ), and amplified (AMT-8, Bortec, Calgary, AB) as needed to maximize the range of the data acquisition system. The analog data were anti-alias filtered at 500 Hz using a 5-pole Bessel filter and sampled at 2.5 kHz (PCI-DAS1602/16, Measurement Computing, Norton, MA, United States). Ankle position was simultaneously recorded using a 24-bit quadrature encoder card (PCI-QUAD04, Measurement Computing, Norton, MA, United States). Data acquisition and motor control were executed using xPC target (The Mathworks Inc., Natick, MA, United States).

### Protocol

Two different experimental sessions were conducted. A unique set of 10 subjects participated in each session. The goal of the first session was to characterize the contribution of impedance and muscle activation to torque generation during movement. The goal of the second session was to evaluate the perceived difficulty of the three tasks performed in both sessions.

The first session began with the collection of maximum voluntary contractions (MVCs) to normalize the recorded EMG and to scale the torque for the later trials (Besomi et al., 2020). A MVC was collected for plantarflexion and dorsiflexion, with the ankle fixed at the neutral posture, set to be 1.75 rad between the shank and the foot. We defined ankle angle to be positive when dorsiflexed from the neutral position, consistent with previous work that has quantified ankle impedance (Mirbagheri et al., 2000). Since the goal of the experiment was to produce torques in the plantarflexion direction, we defined plantarflexion torque to be positive.

**FIGURE 2:**
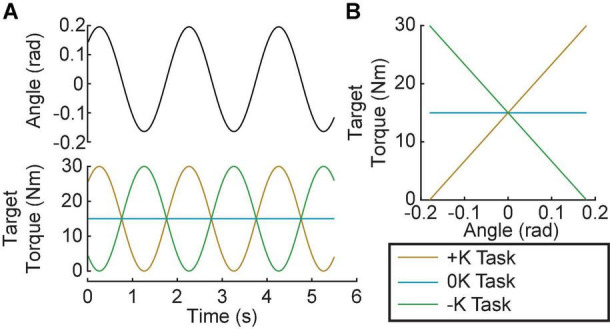
Experimental Protocol. Subjects performed 3 tasks: A positive-quasi-stiffness task (+K); a zero-quasi-stiffness task (0K) and negative-quasi-stiffness task (–K). **(A)** In the +K task the target torque was in phase with the imposed sinusoidal ankle rotation, while it was out of phase for the –K task. **(B)** In the +K task, the slope of the target torque-angle trace is positive, thus we termed it a positive-quasi-stiffness task. Similarly, the slope of the target torque-angle trace is zero in the zero-quasi-stiffness (0K) task, and negative in the negative-quasi-stiffness (–K) task.

**FIGURE 3:**
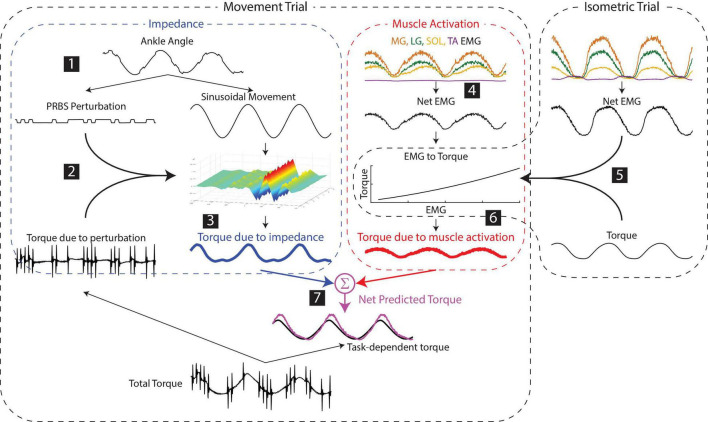
Estimating the contributions of impedance and muscle activation to the net torque about the ankle. (1) Small pseudorandom binary sequence perturbations (PRBS) were superimposed on the larger sinusoidal movement. (2) Ankle impedance was estimated by fitting a time-varying impulse response function between the small perturbation and the resultant torque. (3) The torque due to the impedance was computed by convolving the larger sinusoidal movement with the estimated impedance. (4) The net muscle activity about the ankle was approximated as the difference between the average of the plantarflexor activity and the dorsiflexor activity. (5) The torque due to muscle activation was predicted by a static relationship between the net EMG and ankle torque, estimated from data collected in a separate isometric experiment for each subject. (6) The torque due to muscle activation in the movement trials was computed using this static relationship and the EMGs measured in the movement trial. (7) The net predicted torque was computed as the sum of the torques due to impedance and muscle activation.

Subjects completed three tasks: (1) a positive quasi-stiffness task (+K); (2) a zero quasi-stiffness task (0K); (3) and a negative quasi-stiffness task (–K). The +K task required subjects to produce torques as if they were “resisting” an imposed movement, while the –K task required subjects to produce torques as if they were “assisting” the imposed movement. For all tasks, the actuator moved the ankle through a sinusoidal motion with a frequency of 0.5 Hz and an amplitude of 0.18 rad, centered about the neutral position (Figure 2A). This frequency and amplitude were selected as they are similar to the ankle kinematics during walking (Borghese et al., 1996). For the +K and –K tasks, subjects were instructed to produce a 0.5-Hz sinusoidal plantarflexion torque ranging from 0 to 30% MVC and were aided by visual feedback. The magnitude of the target torque was selected to be feasible without fatigue over the duration of our experiments. For the +K task, the desired torque was in phase with the movement, while for the –K task the desired torque was 180° out of phase with the movement. For the 0K task, subjects were instructed to maintain plantarflexion torque constant at 15%. Maintaining this level ensured that the average torque was consistent across all three tasks. The +K task resulted in an angle-torque relationship with a positive slope (Figure 2B) and hence a quasi-stiffness that was consistent with the impedance of the ankle. In contrast, the 0K and –K tasks had zero and negative slopes, respectively, and could not be achieved simply by altering the mechanical impedance of the ankle. In all tasks, subjects were provided visual feedback of their torque and the target torque trajectory. Prior to beginning each task, subjects were given a minimum of 2 150-s trials (1 without and 1 with perturbations) where they practiced coordinating their torque with the imposed movement. Once subjects were able to track the desired torque with an error that had a standard deviation of less than 3% MVC, we proceeded to the experimental trials. All subjects required only two trials for the +K and –K tasks, but some found the 0K task more difficult. On average 2.6 ± 1.0 trials were required for complete training for the 0K task. We subsequently collected five 150-s trials for each of the three tasks. An additional trial was collected to determine the passive mechanics of the ankle. This involved applying the same sinusoidal movement to the ankle while subjects remained relaxed. A small pseudo-random binary sequence (PRBS) perturbation was imposed on the larger sinusoidal movement to estimate ankle impedance. The PRBS perturbation had an amplitude of 0.035 rad, a velocity of 1.75 rad/s, and a switching time of 0.153 s (Figure 3). Finally, each subject tracked the sinusoidally varying target torque while the ankle position was held constant. The data from this isometric trial was used to estimate the relationship between changes in muscle activation and ankle torque.

Task difficulty was assessed in a separate group of subjects who completed the same three tasks as in the first session, but without superimposed perturbations. Subjects completed one 60-s trial for each task, and the order of the trials was randomized. After completing all trials, subjects assigned a difficulty score to each task using a continuous scale from 1 to 5, with 1 defined as very easy and 5 as very hard.

### Estimating Contributions of Impedance and Muscle Activation to Ankle Torque

**FIGURE 4:**
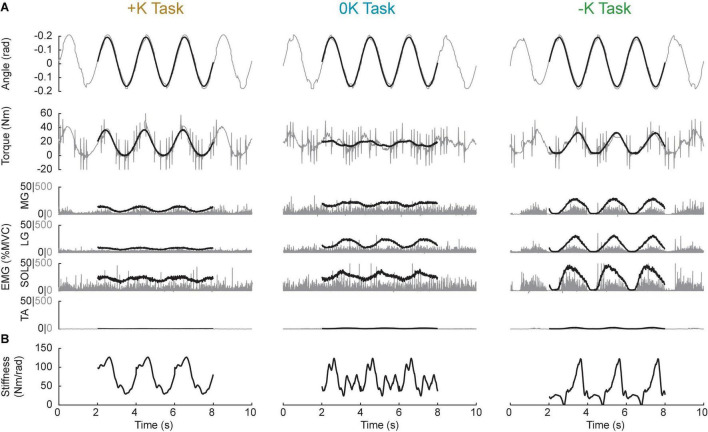
Sample data from a representative subject. **(A)** 10-s snippet of raw data (gray) along with the average of the best 200 segments gathered from the data (black line) for all three tasks. **(B)** Estimates of the stiffness component of impedance throughout the task. Impedance, and hence stiffness, could only be computed with the ensemble of the 200 segments, thus we do not have estimates for a single trial, to match the 10-s snippets shown in **(A)**.

Prior to analysis, the recorded EMGs were notch-filtered to remove 60-Hz noise and full-wave rectified. The angle, torque and rectified EMG signals were digitally filtered to prevent aliasing and decimated to 100 Hz. Ankle impedance was estimated using an ensemble system identification algorithm that requires numerous replications of a repeatable behavior (Ludvig and Perreault, 2012). We therefore segmented all signals into overlapping three-period long segments, with each segment beginning one period (2 s) after the previous one. This resulted in approximately 370 segments for each task. We used the 200 segments with the lowest mean-squared error between measured torque and the target torque to maximize the similarity of our repetitions used for system identification. Finally, torque and EMG were normalized by each subject’s MVC torque/EMG to facilitate comparisons across subjects. Figure 4A shows a 10-s snippet of recorded data from a representative subject, with the average of the 200 segments superimposed.

#### Estimation of Impedance Contributions to Torque

Following this pre-processing, the torque due to impedance was computed as follows (numbers correspond to the steps shown in Figure 3):

1. The large sinusoidal movement and small random perturbations—as well as the torque due to the sinusoidal movement and the random perturbations—were separated by removing the ensemble mean of the ankle angle and torque from each periodic segment.
2. Ankle impedance was estimated by computing a non-parametric, time-varying impulse response function (IRF) at each point within the periodic ankle motion (Ludvig and Perreault, 2012). This impulse response described the relationship between the small PRBS perturbations and the ankle torques opposing them. Two-sided IRFs (Kearney and Hunter, 1990; Westwick and Perreault, 2012) were estimated with a duration ranging from −0.06 to 0.06 s relative each instance in time; the estimation used a 0.1-s window of data centered about this same time point (the mean %VAF of the time-varying IRFs in all subjects and tasks was 81 ± 10%, *n* = 30). The non-parametric IRFs were subsequently parameterized using a second order model consisting of a stiffness, viscosity, and inertia (Ludvig et al., 2011; Ludvig and Perreault, 2012). Figure 4B shows the stiffness estimate of the impedance, which we will show to be the dominant component of the impedance at the frequencies relevant to the completion of the three tasks.
3. The torque due to the impedance (*Tq*_*I*_) was computed by convolving the estimated time-varying IRF (*h(t,t)*) with the imposed sinusoidal movement (*q*).
  (1) $$Tq_{I}\left(t\right)=\int \int_{0}^{t}h\left(t,\tau \right)\frac{d\theta }{dt}\left(t-\tau \right)d\tau dt$$

This time-varying convolution allows for the impedance to vary non-linearly with torque or ankle angle, and assumes an initial equilibrium position at 0, the center angle of the imposed movement. This computation of impedance torque is insensitive to changes in the equilibrium position throughout the movement. The validity of this assumption, which allows us to distinguish torque due to impedance from that due to muscle activation, is assessed by evaluating the accuracy of the modeled torque across all experimental conditions.

This procedure was done for all three tasks, as well as the data collected in the passive trial.

#### Estimation of Muscle Activation Contributions to Torque

Following the initial EMG pre-processing outlined above, the torque due to muscle activation was computed as follows:

4. For all tasks, the net EMG was computed by computing the difference between the average plantarflexor (LG, MG, SOL) EMG activity and the dorsiflexor (TA) EMG activity.
5. A 2nd order polynomial was fit between the net EMG and the torque measured in the isometric task (%VAF = 98.0 ± 0.8%, *n* = 10) to create a model of the EMG to torque relationship.
6. The torque due to muscle activity during the movement trials was predicted from the EMGs measured in these trials and the isometric model.
7. The net predicted torque was computed by summing the torque due to muscle activity with the torque due to impedance computed in step 3. It is important to note that the models used to predict torques due to both impedance and muscle activation were estimated from data separate from that on which the full model was evaluated. The torque response to small perturbations was used to estimate impedance, and isometric contractions were used to estimate the EMG-torque relationship. We then used these estimated models to predict the net torque during each of the three tasks involving sinusoidal movements (Figure 3).

### Evaluation of Task Performance

Task performance was evaluated by how well subjects matched the target torque, how consistent they were from trial to trial, and whether they had any consistent deviations from the target. Overall performance was quantified by the total error, which described how well the subjects followed the desired torque trajectory. It was computed by the root mean square (RMS) of the tracking error between desired and actual torque trajectories. This total error was then broken down into two components: random error and bias error. Random error was a measure of consistency, as it quantified how much trial-to-trial variability there was in the torque trajectories. It was computed by finding the RMS of the torque trajectories after removing the average torque trajectory. Finally, the bias error, was used to quantify consistent deviations from the target torque. It was computed by finding the RMS of error between the desired and average torque trajectories.

### Statistical Analysis

The goal of this study was to determine how leveraging joint impedance when feasible simplified the neural control of movement. Specifically, we tested the hypothesis that tasks that leveraged the impedance of the ankle would be completed with an easier perceived difficulty and consistently. We compared three metrics across the three tasks that were studied: the torque due muscle activation, perceived difficulty across the tasks, and performance in each of the three torque-tracking tasks. We used a repeated measures ANOVA to test for differences in each of these metrics across the three tasks. *Post hoc* analyses were computed using Tukey’s Honest Significant Difference when needed. Additionally, we ran paired *t*-tests to determine whether impedance or muscle activation was greater in each task. For all tests, significance was set to *p* = 0.05. Results are presented as the mean and 95% confidence intervals (mean ± 47.5% confidence interval), unless otherwise specified. We completed the data analysis in MATLAB (2017a, MathWorks).

## Results

### A Model of Torques Due to Impedance and Muscle Activation Described Experimental Data

**FIGURE 5:**
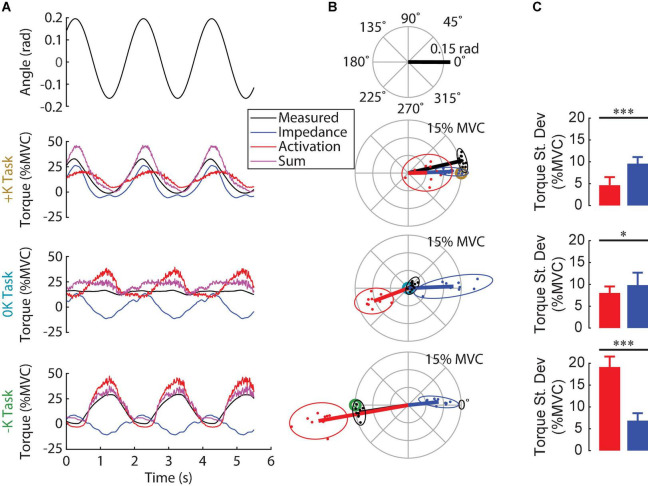
Contribution of impedance and muscle activation to the torque generated in the three tasks. **(A)** Ankle angle, measured torque, torque due to impedance, torque due to muscle activation and their sum for all three tasks for one subject. The sum of the two modeled torque components was a good fit for the measured torque for this subject in all three tasks. **(B)** Polar plot showing the phase and magnitude of the different torque components for all subjects. For each task, the target torque is denoted by a bullseye. Since the torque due to impedance was always in phase with the imposed movement, muscle activation was required to compensate for the impedance when it was not beneficial to task performance. **(C)** The torque due to impedance was greater than the torque due to muscle activation in the +K task, whereas these torques were of similar magnitude in the 0K task, and had a reversed order of dominance in the –K task (*p < 0.05, ^**^p < 0.01; ^***^p < 0.001). We conclude that subjects completed the +K task by relying more on ankle impedance and reducing muscle activity compared to the other two tasks.

We found that the experimentally measured ankle torque was modeled well by our simple model predicting the torques due to impedance and muscle activation. Figure 5A shows the experimentally measured torque, the predicted torques due to impedance and muscle activation, and the net predicted torque (impedance + muscle activation) for a typical subject. Across all movement trials, the standard deviation of the residual error of this model was 4.0 ± 1.6% MVC (mean ± S.D.; *n* = 30). This was a rather small error relative to the large torques produced in certain movement trials that were up to 30% MVC. Furthermore, these errors were consistent across the three tasks [+K: 3.6 ± 0.8% MVC, 0K: 4.2 ± 1.0% MVC, –K: 4.3 ± 1.5% MVC; *F*_(2_, _18)_ = 0.58, *p* = 0.58]. These results suggest that the assumptions inherent in the model are appropriate for the tested experimental conditions. Separating the measured torque into these two components allowed us to investigate how these two potential mechanisms for regulating motion-torque coordination were employed in each of the tested tasks.

### Tasks That Leverage Limb Impedance Reduce the Need for Muscle Activation

We examined the contributions of ankle impedance and changes in muscle activation to the net torque at the ankle to determine the strategies that subjects employed in each task. Figure 5A shows the measured torque, the torques attributed to the impedance and muscle activation and the sum for a representative subject. Figure 5B shows the magnitude and the phase of the 0.5 Hz component of these torques for all subjects. For both the representative subject and the entire group, the torque due to impedance closely matched the measured torque in the +K task, where subjects produced torques “resisting” the imposed movement. For this task, the impedance torque accounted for 88 ± 5% (mean ± S.D.; *n* = 10) of the measured torque variance. In contrast, in the 0K task, the torque from impedance was a sinusoid of similar magnitude to the +K task, but not helpful as the 0K task required no sinusoidal torque component. Finally, in the –K task, where subjects produced torques to “assist” the imposed movement, the torque from impedance was a sinusoid out of phase with the measured torque, and therefore counterproductive.

**FIGURE 6:**
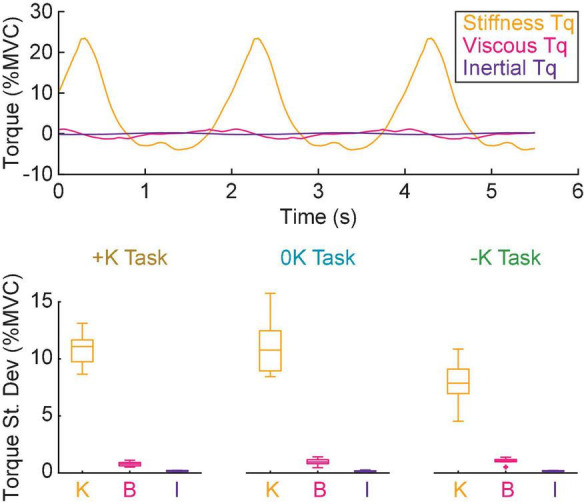
Stiffness was the dominant contributor to the impedance torque. **(A)** The torque due to, stiffness (orange) viscosity (magenta) and inertia (purple) for a single task in a representative subject. **(B)** The torque due to stiffness was approximately an order of magnitude greater than the torque due to viscosity and two orders of magnitude greater than the torque due to inertia for all tasks.

In all tasks, the torque due to impedance was dominated by the stiffness, resulting in an impedance torque that was in phase with the movement. The non-parametric IRFs quantifying ankle impedance were parameterized by second-order models with stiffness, viscosity, and inertia (Figure 6). The torque due to stiffness (+K: 10.9 ± 1.1% MVC; 0K: 11.2 ± 1.8% MVC; –K: 7.9 ± 1.4% MVC) was an order of magnitude greater than the torque due to viscosity (+K: 0.79 ± 0.14% MVC; 0K: 0.96 ± 0.20% MVC; –K: 1.06 ± 0.16% MVC) and two orders of magnitude greater than the torque due to inertia (0.16 ± 0.04% MVC in all tasks) in all three tasks.

Subjects used changes in muscle activation to compensate for counterproductive impedance torques. Since for all tasks, the torque from ankle impedance was in phase with the movement (Figure 5B), the presence or timing of the impedance torque was therefore counterproductive in the 0K and –K tasks, respectively. This required subjects to compensate for these counterproductive torques in the 0K and –K tasks through changes in muscle activation. The consequence was that subjects produced less cyclic torque from changes in muscle activation during the +K task and more torque due to ankle impedance (Δ = 4.9 ± 2.1% MVC, t_9_ = 5.3, *p* = 0.0005) (Figure 5C). These contributions to the net cyclic ankle torque were more comparable in the 0K task (Δ = 1.8 ± 1.5% MVC, t_9_ = 2.8, *p* = 0.02), while there was greater cyclic torque due to muscle activation in the –K Task (Δ = −12.3 ± 1.6% MVC, t_9_ = −17.5, *p* < 0.0001). Together, these results suggest that subjects relied more heavily on the impedance of the ankle to meet the task demands when impedance was consistent with the task requirements.

**FIGURE 7:**
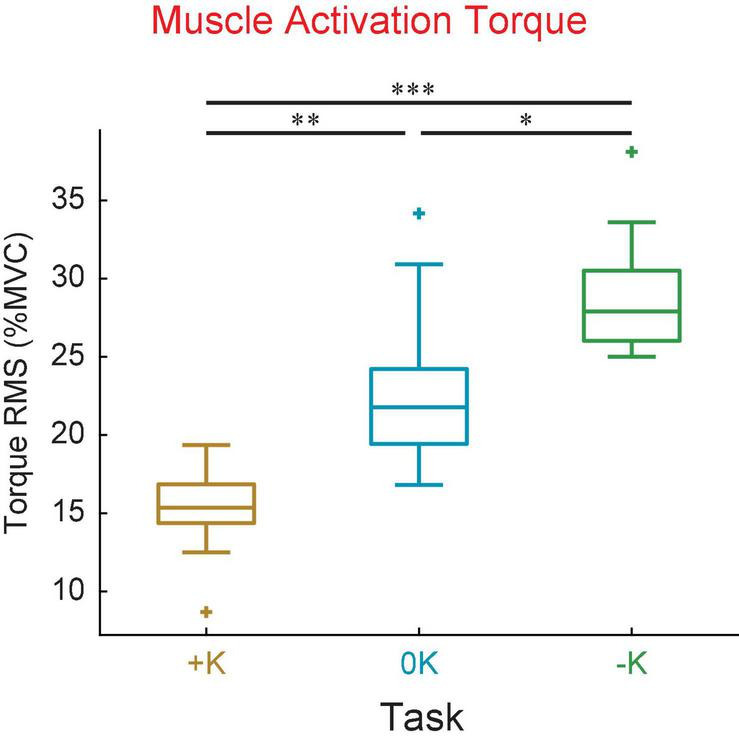
The +K task required subjects to generate less torque due to muscle activation compared to the other two tasks. This was seen both in the mean torque as well as the standard deviation of torque about the mean value. *p < 0.05; **p <0.01; ***p < 0.001.

We compared the torque from muscle activation across the three tasks (Figure 7), to confirm our expectation that tasks which aligned with the impedance of the joint would require less muscle activation. We found that the torque due to muscle activation was smallest in the +K task. The torque due to muscle activation, as measured by the root mean square, varied between the different tasks [*F*_(2_, _18)_ = 27, *p* < 0.0001]. The torque from muscle activation was significantly lower in the +K task compared to the –K task (Δ = 13.9 ± 4.9% MVC, *p* < 0.0001) and the 0K task (Δ = 7.9 ± 4.9% MVC, *p* = 0.002). These results confirm our expectation that the +K task resulted in a task which required less torque due to muscle activation, allowing us to link any changes in perceived difficulty and task performance to a decreased reliance on neurally controlled muscle activation.

To rule out the possibility that co-contraction resulted in an increase in muscle activation with no increase in joint torque, we verified that the plantarflexor EMG activity was lower in the +K task. We found that the mean plantarflexor EMG was significantly lower in the +K task compared to the 0K task (Δ = 5.8 ± 3.2% MVC, *p* = 0.0005) or the –K task (Δ = 4.4 ± 3.2% MVC, *p* = 0.006). This lesser amount of plantarflexor EMG in the +K task indicates that co-contraction of the plantarflexors and dorsiflexors did not result in low levels of torque due to muscle activation despite high levels of muscle activity.

### Tasks That Can Leverage Limb Impedance Are Perceived as Easy to Perform

**FIGURE 8:**
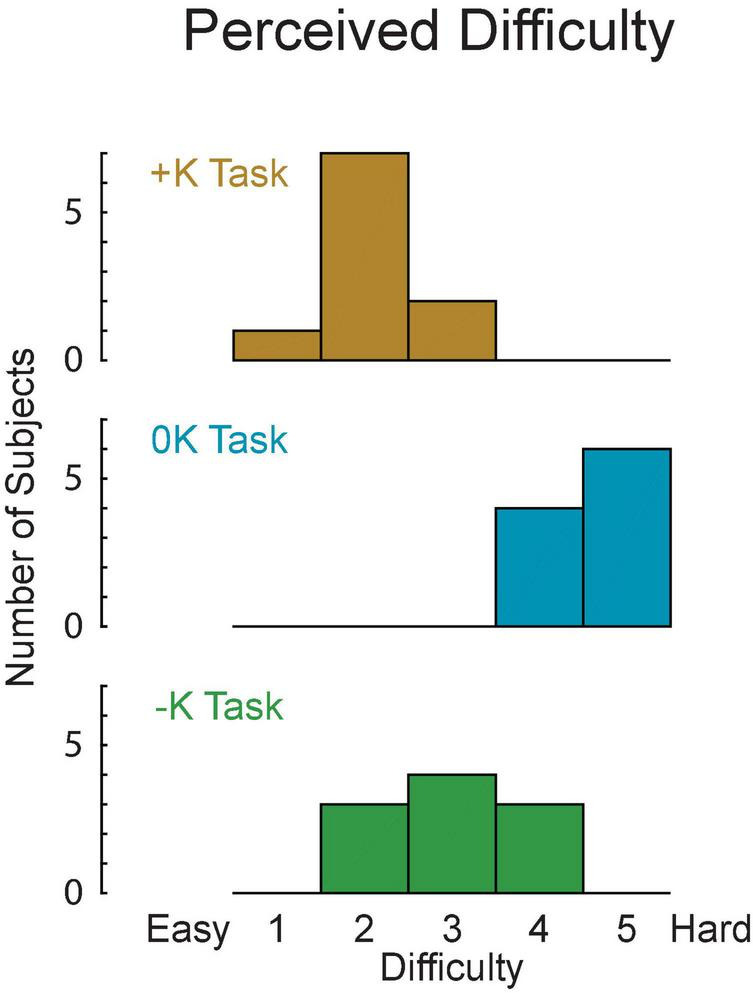
The +K task was perceived to be easier by the subjects, as rated on a 1–5 difficulty scale.

We asked subjects to rate the difficulty of each task to determine how the different strategies we observed changed the perception of task difficulty. Difficulty was rated, from 1 to 5 on a continuous scale. These subjective measures were obtained from a new set of subjects so that previous exposure to the three torque-tracking tasks did not alter perceived difficulty. Eight of the 10 subjects perceived the +K task to be easiest, while 2 subjects found the –K task to be easiest. All subjects found the 0K task to be most difficult. Using the subjects’ reported perceived difficulty scores (Figure 8), we found that the there was a significant perceived difficulty between the three tasks [*F*_(2_, _18)_ = 38, *p* < 0.0001]. The +K task was found to be significantly easier than both the –K (Δ = 1.0 ± 0.7, *p* = 0.009) and the 0K tasks (Δ = 2.5, ± 0.7, *p* < 0.0001).

### Tasks That Leverage Limb Impedance Can Be Performed More Consistently Than Others

**FIGURE 9:**
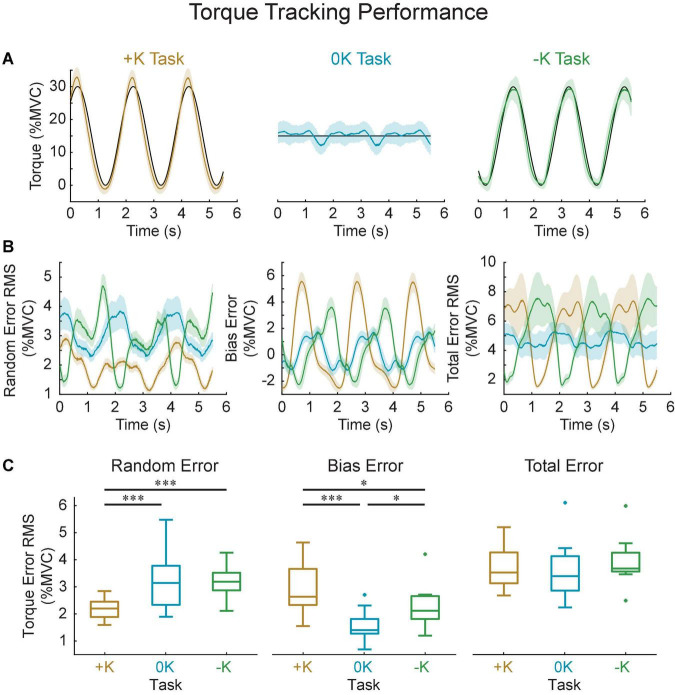
Task performance was quantified by how well the measured torque matched the torque target in all three tasks. **(A)** Representative data for one subject, showing the target (black), the average measured torque (colored lines) and standard deviation across all the cycles of the movement (shaded area). **(B)** Computed random, bias and total error and their variation throughout the imposed movement. Solid shows group average, shaded area shows 95% confidence interval of the group average. **(C)** While the total error did not differ between the three tasks, the +K task did show less random error, indicating a more consistent task behavior when subjects could rely more heavily on impedance. *p < 0.05; ***p < 0.001.

We assessed the torque-tracking errors to evaluate how the different control strategies influenced performance in each task. Specifically, we quantified the total, random, and bias tracking errors for the original groups of subjects (Figure 9) in which we estimated the impedance from. Both random and bias error varied cyclically with the imposed movement, resulting in a total torque error that was approximately proportional to the torque applied by each subject. As a result, we saw no difference in the total tracking error across tasks [*F*_(2_, _18)_ = 1.6, *p* = 0.223]. This similar performance may be due to fact that subjects were trained to achieve a certain level of proficiency in matching the torque. However, we did observe differences in random error [*F*_(2_, _18)_ = 16, *p* = 0.0001], which assesses the consistency of task performance across repeated cycles of movement. There was significantly less random error in the +K task compared to the –K (Δ root mean square error = 1.0 ± 0.5% MVC, *p* = 0.0004) and 0K tasks (Δ = 1.0 ± 0.5% MVC, *p* = 0.0003). We also saw differences in the steady state or bias error across repeated movements in each of the three tasks [*F*_(2_, _18)_ = 20, *p* < 0.0001]. There was greater bias error in the +K task compared to the –K (Δ = 0.7 ± 0.5% MVC, *p* = 0.01) and 0K tasks (Δ = 1.4 ± 0.5% MVC, *p* < 0.0001).

**FIGURE 10:**
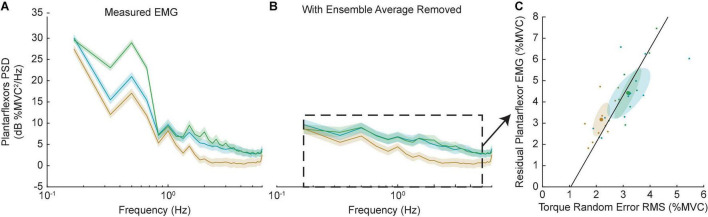
Random error was correlated with increased cycle-to-cycle muscle activity variability. **(A)** Power spectra of the plantarflexor EMGs for all three tasks. Solid line shows group average, shaded areas show 95% confidence internals. As expected, the –K task had the greatest EMG activity. **(B)** Removing the ensemble average allowed us to investigate the EMG activity that differed from cycle-to-cycle. The +K task had less EMG than the other two tasks at all frequencies. **(C)** This residual EMG correlated strongly with the amount of random torque error across all subjects and tasks (black line). As a result, in the +K task subjects had less random torque error and less cycle-to-cycle variability in their EMGs.

The decrease in random error in the +K task may suggest a neural control strategy that required fewer cycle-to-cycle corrective actions by the subject. We assessed this possibility by computing the cycle-to-cycle variation in the plantarflexor muscle activity, which was quantified by the power of plantarflexor EMG following removal of the ensemble mean (Figure 10). Similar to the random error, the +K task had the lower cycle-to-cycle variability in EMG compared to both the 0K (Δ = −1.2 ± 0.9% MVC, *p* = 0.005) and –K tasks (Δ = −1.3 ± 0.9% MVC, *p* = 0.005), while the –K and 0K task had similar amounts of variability (Δ = 0.0% ± 0.8% MVC, *p* = 0.99). Across all subjects we see a strong correlation between the random error and the cycle-to-cycle EMG variability (*r* = 0.78, *p* < 0.0001; Figure 10C), indicating that the increased cycle-to-cycle tracking errors in the –K and 0K tasks were associated with increased variability in how subjects controlled muscle activation across cycles of movement. The reduced need for cycle-to-cycle changes in neural control could be another way in which leveraging the impedance in the +K task resulted in a simpler task to complete.

## Discussion

The purpose of this study was to determine if leveraging the impedance of a limb results in an easier and more consistent task performance. We had subjects complete three torque-tracking tasks using their ankle, only one of which could be achieved by leveraging their impedance (the +K task). We evaluated the control strategy used in each task by estimating the contributions of ankle impedance and muscle activation to the net torque that was produced. We found that subjects generated less torque from muscle activation in the +K task, the task in which subjects had to produce torques to “resist” the imposed movement. Subjects perceived the +K task to be easiest and were able to complete it more consistently than the other two tasks. These differences were not simply the result of the reduced muscle activation required to complete the +K task, as the task that required the most muscle activity was not perceived as most difficult, nor did it exhibit the lowest performance. These results demonstrate that when subjects can leverage joint impedance to complete a motor task, it results in a strategy that is easier and more consistent to perform.

### Tasks That Leverage Limb Impedance Reduce the Need for Muscle Activation

In the +K task when the ankle impedance was consistent with the task demands, subjects produced more torque via ankle impedance and less through muscle activation. While this finding on its own was not too surprising it provided a confirmation of expectations, and a means to quantify how much impedance could help or hinder neural control. In contrast, both other tasks were completed by increasing the torque arising from muscle activation, as neither could be completed using only impedance modulation. This is because the dominant component of impedance in these tasks was stiffness, which is a finite positive value (Hunter and Kearney, 1982) and generates a torque that resists the imposed sinusoidal motion. Generating a constant level of torque (0K task) or a torque that assists the imposed motion (–K task) requires muscle activation to counter the torque due to impedance. Our results agree with this expectation, as there were nearly equivalent torques from impedance and changes in muscle activation in the 0K task and substantially more torque due to muscle activation in the –K task.

Even though subjects could have completed the +K task by setting their impedance to a value appropriate for generating the target torque, few used only this strategy. Completing the +K task through impedance modulation alone would have required our subjects to set their ankle stiffness to constant values ranging from 49 to 94 Nm/rad, depending on each individual’s MVC. This is well within the range of achievable ankle stiffnesses (Hunter and Kearney, 1982; Loram and Lakie, 2002; Loram et al., 2007). Subjects were able to rely on their impedance to generate the majority of the necessary torque as the impedance torque accounted for 88% of the measured torque. However, they did not rely exclusively on an impedance control strategy, as the impedance torque was on average 2.6 times greater than the muscle activation torque (Figure 5C). Thus, while subjects did rely on their impedance to complete the +K task, they did not use an impedance control strategy exclusively.

### Tasks That Leverage Impedance Are Perceived to Be Easier

Subjects perceived the +K task to be the easiest to complete. Two factors have been associated with the perceived difficulty in completing force or torque production tasks: physical and psychological factors (Slobounov et al., 2004). The leveraging of impedance in the +K task resulted in less muscle activation torque and that may partly explain why most subjects found this task easier to perform. However, that explanation is not consistent with the results of the 0K and –K tasks; subjects universally found the 0K task to be most difficult despite that it required less muscle activation torque than the –K task. Therefore, the perceived difficulty may be related more to psychological factors. The verbal feedback provided by the subjects demonstrates this decreased mental challenge in completing the +K task, as several described this task as “easier” or “more natural.” The strategies they employed also suggest a simpler approach to completing the +K task, in which some subjects found it easy to “hold their foot still” in the +K task as opposed to needing to “find the rhythm” in the 0K and –K tasks. However, it remains unknown as to why subject perceived the 0K task to be the most difficult.

### Tasks That Leverage Impedance Are Completed More Consistently

Subjects completed the +K task more consistently than the other two tasks. While overall performance was similar in all three tasks, subjects generated torques that were more consistent from cycle-to-cycle in the +K task. Some of this decreased variability might be explained by signal dependent noise. Muscle activation is an inherently noisy process (Carlton et al., 1985; Harris and Wolpert, 1998; Jones et al., 2002; Tracy et al., 2005) and thus the tasks that require more muscle activation would have more variability. Signal dependent noise could explain the decreased random error in the +K task, but it cannot explain the similar levels of random error in the 0K and –K tasks since the –K task had higher levels of muscle activity than the 0K task (Figures 7, 10A).

The cycle-to-cycle variability in matching the torque target (i.e., the random error) correlated with the variability in muscle activation across cycles. This correlation suggests that subjects were generating corrective bursts of muscle activity in response to deviating from the target torque trajectory (Hu et al., 2017), and that these corrections were largest for the 0K and –K tasks. This suggestion of increased cycle-to-cycle corrections is consistent with the finding that steady-state or bias errors were largest for the +K task. These differences in error performance across tasks demonstrates how leveraging impedance can simplify neural control. Once an appropriate level of limb impedance is established, changes in neural control can be minimized as long as the interaction torques required for task completion remain consistent over time.

### Implications

The mechanical impedance of human limbs has been studied extensively in the context of maintaining stability during postural control (Hogan, 1984; Mussa-Ivaldi et al., 1985; De Serres and Milner, 1991; Trumbower et al., 2009; Krutky et al., 2013) and movement (Gomi and Kawato, 1996; Burdet et al., 2001; Franklin et al., 2007; Zenzeri et al., 2014). When stability is compromised by unexpected disturbances or the exertion of forces on the environment, we are able regulate impedance so as to complete the task at hand (Hogan, 1985a). Impedance regulation has also proven to be an important concept for robot control (Anderson and Spong, 1988; Vanderborght et al., 2013), as it can be used to generate stable motions and postures along with reliable and forceful contact with the environment. Impedance in robotics has been implemented both through software (Semini et al., 2015) and hardware (Pratt and Williamson, 1995; Vanderborght et al., 2013), analogous to the roles of muscle activation and joint impedance play in generating torques in our study, respectively. Using a hardware based approach can be simpler as it reduces the complexity of the required control algorithms (Vanderborght et al., 2013). We believe that our experimental findings are the first to demonstrate that humans can also leverage the impedance of their limbs to simplify the control required to generate forceful interactions with the environment.

Many common locomotor tasks require joint motion-torque relationships, or quasi-stiffnesses, that are consistent with the impedance of our limbs. Due to the ability of our central nervous system to precisely control muscle activation, humans can generate a variety of motion-torque patterns at each joint. One behavior that arises in many lower-limb movements is a spring-like behavior, or a positive quasi-stiffness, for which the generated joint torques or limb forces act to oppose changes in length. Positive quasi-stiffness can be seen at the whole limb level (Blickhan, 1989; Ferris and Farley, 1997) and at the joint level (Farley and Morgenroth, 1999; Shamaei et al., 2013a) during human locomotion. For example, the ankle, knee, and hip exhibit positive quasi-stiffness in a variety of tasks including, walking (Shamaei et al., 2013a,b, c; Rouse et al., 2014), running (Stefanyshyn and Nigg, 1998; Arampatzis et al., 1999; Kuitunen et al., 2002), and hopping (Farley and Morgenroth, 1999). This positive quasi-stiffness is also present at the muscle-tendon level during many phases of animal locomotion (Tu and Dickinson, 1996; Roberts et al., 1997; Biewener et al., 1998). A simple and efficient way to achieve this quasi-stiffness would be to match the impedance of the joint to the behavioral demands so that the demands on changes in muscle activation are minimized. Our results demonstrate that task-appropriate impedance simplifies neural control. An important complementary experiment would be to evaluate if impedance is actively regulated to simplify neural control.

### Limitations

Our results were obtained using a novel method for decomposing the net torque about the ankle into components arising from impedance and from muscle activation. This was useful for estimating the relative contributions of these two strategies for controlling the net torque about the ankle, but potential errors in the estimation process should be considered. However, it is important to note that any errors in estimating either torque due to impedance and muscle activation would not alter our primary conclusions that the +K task was completed more consistently and perceived as easier to perform, as these primary outcomes were independent of our decomposition technique.

The model used to estimate the torque from muscle activation was constructed from data measured during isometric contractions rather than the cyclical movements in which it was eventually used. We chose to use an isometric model to estimate the EMG-torque relationship to limit the assumptions made and to avoid directly fitting models to the data. Directly fitting the data would require us to remove the contribution of the impedance from the measured torque, and any errors in our estimation of the impedance torque could bias our estimates of the torque due to muscle activation. While using isometric data to determine the EMG-torque relationship has the advantage of not being confounded by impedance torque it did have limitations. Specifically, there was little activity in the dorsiflexor muscle during the isometric trial and the EMG-torque relationship changes during movement, as this relationship is sensitive to the angle of the joint (Liu et al., 2013, 2015) and the velocity of movement (Wickiewicz et al., 1984). These effects may have introduced errors in our predictions of muscle activation torque during our movement conditions, but the residuals of our model suggest that these errors were modest and consistent across all tested conditions. There was little activity in the dorsiflexor muscles during the three cyclic movement tasks, thus inaccuracies in modeling the contribution of the dorsiflexor would not greatly affect our prediction of the torque due to muscle activation. As can be seen in the sample data shown in Figure 4, the muscle activity in the dorsiflexor muscle (TA), is substantially smaller than the activity in the plantarflexor muscles for all tasks. Across all subjects, the average activity in the plantarflexor muscles was 11 ± 2, 10 ± 2, and 6 ± 1 times greater than the activity in the dorsiflexor muscles in the +K, 0K, and –K tasks, respectively. Thus, any errors in modeling the torque due to the dorsiflexor muscles would be overshadowed by the much larger torques generated by the plantarflexor muscles. We further determined error bounds, by directly fitting a model between the EMG and torque following removal of the impedance. This model, which was directly fit to the data, did predict less torque due to muscle activation compared to the isometric model we used for our main results, but this decrease in predicted torque due to muscle activation was consistently lower in all tasks [+K: 5 ± 3% MVC; 0K: 7 ± 3% MVC; –K: 7 ± 3% MVC; *F*_(2, 18)_ = 1.0, *p* = 0.37], and thus would not have affected our conclusions.

Our estimates of impedance were obtained during cyclical movements, but the use of these estimated to compute the torque due to impedance relied on an important assumption. This was that any changes in torque arising from changes in the set-point or equilibrium position were captured by the muscle activation torque component. The accuracy of our modeled torque, which was not fit to the experimental measures, suggests that any errors in the estimated impedance torque were modest (4.0% MVC). In addition, it is important to note that any errors that did exist would not alter our primary conclusions that the +K task was completed more consistently and perceived as easier to perform, as these primary outcomes were independent of our decomposition technique.

Our study only focused on leveraging joint mechanics in the ankle to simplify neural control. It is remains unknown if our findings generalize to other joints, especially to those of the upper limb. Different brain areas are known to control the upper and lower limbs (Luft et al., 2002). While impedance control has been shown in both upper (Krutky et al., 2013) and lower limbs (Finley et al., 2012), we do not know if our specific findings generalize across limbs.

## Conclusion

In summary, we found that humans can leverage the impedance of the ankle to simplify neural control when that impedance is consistent with the motion-torque demands of the task. Such a strategy reduced the required muscular effort, leading to performance that was perceived to be easier and was completed more consistently. These findings were enabled by the novel method we developed that allowed us to separately estimate the contributions of ankle impedance and changes in neural control to the net ankle torque during large movements. These results suggest that relying on impedance could be a simple way to complete many tasks that require spring-like motion-torque profiles from the joints within the human body.

## Data Availability Statement

The raw data supporting the conclusions of this article will be made available by the authors, without undue reservation.

## Ethics Statement

The studies involving human participants were reviewed and approved by the Northwestern University Institutional Review Board. The patients/participants provided their written informed consent to participate in this study.

## Author Contributions

DL and MW collected and analyzed the data. DL and EP interpreted the results. DL, MW, and EP drafted the manuscript, while DL generated all the figures. All authors have approved the final version of the manuscript and have agreed to be accountable for all aspects of the work and contributed to the conceptual design of the study, and confirm that all listed authors qualify to be listed as authors, and that no one else qualifies to be an author of this manuscript.

## Conflict of Interest

The authors declare that the research was conducted in the absence of any commercial or financial relationships that could be construed as a potential conflict of interest.

## Publisher’s Note

All claims expressed in this article are solely those of the authors and do not necessarily represent those of their affiliated organizations, or those of the publisher, the editors and the reviewers. Any product that may be evaluated in this article, or claim that may be made by its manufacturer, is not guaranteed or endorsed by the publisher.
